# A Text Book of Obstetrics

**Published:** 1890-04

**Authors:** 


**A Text-Book of Obstetrics,** including the Pathology and Therapeutics of the Puerperal State. Designed for Practitioners and Students of Medicine. By Dr. F. WINCKEL, Professor of Gynecology and Director of the Royal Hospital for Women; member of the Supreme Medical Council, and of the Faculty of Medicine in the University of Munich. Translated from the first German edition under the supervision of J. Clifton Edgar, A. M., M. D., Adjunct Professor of Obstetrics in the Medical Department of the University of New York. 190 illustrations. Philadelphia: P. Blakiston, Son & Co., 1012 Walnut street. 1890.

The present work is a valuable contribution to the literature of obstetrics. Few writers have such an inexhaustible store-house of experience from which to draw in the preparation of a suitable text-book for students and practitioners. We think Dr. Winckel has made good use of the material at hand, and also presented the matter in a form to benefit the class of readers to whom he especially directs his attention. In his preface the author states that the "material upon which this book is based" includes cases seen in his own private practice, as well as that of his father and grandfather, and embraces a series of more than 20,000 labors, over 600 of which were purely operative, and 17,200 were taken from his own clinics since 1864.

It may be expected that the work would be eminently practical, while the professional attainments of the author would lend to it a highly scientific character.

The author divides the work into eight parts, the first part being devoted to the physiology and management of pregnancy; the second to the physiology and management of labor; the third, the physiology and management of the puerperal state; the fourth, the pathology and therapeutics of pregnancy; the fifth, the pathology and therapeutics of labor; the sixth, obstetric operations; the seventh, the pathology and therapeutics of the puerperal state; the eighth, the pathology and therapeutics of diseases of the new-born.

Under these "Parts," the author divides the subject into many sections, and thus presents a very methodical and lucid exposition of the science and art of obstetrics, which will assist the accoucheur in his practical work, and also the students in securing a clear, comprehension of this important department of medical science.

It may be stated that the publishers, in presenting to the profession this translation of an important work, have done a valuable service, for which a recognition will surely follow, in its appreciation by both practitioners and students, for whom it is designed.

